# Mechanistic Insight Into the Activation of the NLRP3 Inflammasome by *Neisseria gonorrhoeae* in Macrophages

**DOI:** 10.3389/fimmu.2019.01815

**Published:** 2019-07-31

**Authors:** Lan-Hui Li, Jia-Sing Lin, Hsiao-Wen Chiu, Wen-Yu Lin, Tz-Chuen Ju, Fang-Hsin Chen, Oleg V. Chernikov, May-Lan Liu, Jen-Che Chang, Chung-Hua Hsu, Ann Chen, Shuk-Man Ka, Hong-Wei Gao, Kuo-Feng Hua

**Affiliations:** ^1^Department of Laboratory Medicine, Linsen Chinese Medicine and Kunming Branch, Taipei City Hospital, Taipei, Taiwan; ^2^Department of Pathology, National Defense Medical Center, Tri-Service General Hospital, Taipei, Taiwan; ^3^National Defense Medical Center, Graduate Institute of Life Sciences, Taipei, Taiwan; ^4^Division of Cardiology, Department of Internal Medicine, National Defense Medical Center, Tri-Service General Hospital, Taipei, Taiwan; ^5^Department of Animal Science and Biotechnology, Tunghai University, Taichung City, Taiwan; ^6^Department of Medical Imaging and Radiological Sciences, Chang Gung University, Taoyuan City, Taiwan; ^7^G.B. Elyakov Pacific Institute of Bioorganic Chemistry (PIBOC), Far Eastern Branch of the Russian Academy of Sciences (FEB RAS), Vladivostok, Russia; ^8^Department of Nutritional Science, Toko University, Chiayi City, Taiwan; ^9^School of Medicine, Institute of Traditional Medicine, National Yang-Ming University, Taipei, Taiwan; ^10^Department of Medicine, National Defense Medical Center, Graduate Institute of Aerospace and Undersea Medicine, Taipei, Taiwan; ^11^Department of Biotechnology and Animal Science, National Ilan University, Yilan City, Taiwan; ^12^Department of Medical Research, China Medical University Hospital, China Medical University, Taichung City, Taiwan

**Keywords:** *Neisseria gonorrhoeae*, NLRP3 inflammasome, macrophages, signal transduction, bactericidal activity

## Abstract

Gonorrhea is a type III legal communicable disease caused by *Neisseria gonorrhoeae* (NG), one of the most common sexually transmitted bacteria worldwide. NG infection can cause urethritis or systemic inflammation and may lead to infertility or other complications. The NACHT, LRR, and PYD domains-containing protein 3 (NLRP3) inflammasome is a protein complex composed of NLRP3, apoptosis-associated speck-like protein and caspase-1 and is an important part of the cellular machinery controlling the release of interleukin (IL)-1β and IL-18 and the pathogenesis of numerous infectious diseases. It has been reported that NG infection activates the NLRP3 inflammasome; however, the underlying mechanism remain unclear. In this report, the signaling pathways involved in the regulation of NG-mediated NLRP3 inflammasome activation in macrophages were studied. The results indicated that viable NG, but not heat-killed or freeze/thaw-killed NG, activated the NLRP3 inflammasome in macrophages through toll-like receptor 2, but not toll-like receptor 4. NG infection provided the priming signal to the NLRP3 inflammasome that induced the expression of NLRP3 and IL-1β precursor through the nuclear factor kappa B and mitogen-activated protein kinase pathways. In addition, NG infection provided the activation signal to the NLRP3 inflammasome that activated caspase-1 through P_2_X_7_ receptor-dependent potassium efflux, lysosomal acidification, mitochondrial dysfunction, and reactive oxygen species production pathways. Furthermore, we demonstrated that NLRP3 knockout increased phagocytosis of bacteria by macrophages and increases the bactericidal activity of macrophages against NG. These findings provide potential molecular targets for the development of anti-inflammatory drugs that could ameliorate NG-mediated inflammation.

## Introduction

According to the World Health Organization (WHO) estimation, there were 78.3 million global new gonorrhea cases in 2012, with the largest number of new cases occurring in the low- to middle-income Western Pacific (1). According to reports from the Taiwan Centers for Disease Control (Taiwan CDC), there were 4,469 reported gonorrhea cases in 2016, an increase of approximately 1,000 cases annually from 2013 to 2016 in Taiwan. The emergence of resistance and reduced drug susceptibility in *Neisseria gonorrhoeae* (NG) strains that cause gonorrhea are major health concerns (2). Broad and inadequate uses of antibiotics, gene transfer, and recombination have led to the development of resistance in NG. Because third-generation cephalosporin is recommended as a first-line treatment, close monitoring of the susceptibility of NG to cephalosporin is needed in Taiwan (3).

The clinical pathogenesis of acute gonorrhea is coordinated by macrophages, dendritic cells, neutrophils, T cells, epithelial cells and cytokines in the reproductive tract (4). Although gonorrhea is curable, infection with NG can cause cervicitis, urethritis, proctitis and other pelvic inflammatory diseases, leading to infertility, ectopic pregnancy, chronic pelvic pain, and potentially increased susceptibility to human immunodeficiency virus infection (5). Pathogen-associated molecular patterns (PAMPs) of NG, including lipooligosaccharide, peptidoglycan fragments, porin and lipoproteins, elicit host immune responses and inflammation (4). During infection, toll-like receptor 4 (TLR4) and toll-like receptor 2 (TLR2) recognize gonococcal PAMPs and consequently activate the downstream inflammatory signaling cascade in immune cells (6). In addition to the TLRs located in the plasma membrane and endosomes, intracellular nucleotide binding oligomerization domains (NOD)-like receptors (NLRs) also sense a variety of gonococcal PAMPs to initiate appropriate immune responses in hosts (7). There are more than 20 members of the NLR family that have been identified in humans, and different NLRs sense various danger insults (8). For example, NACHT, LRR, and PYD domains-containing protein 3 (NLRP3) responds to a range of cytosolic stimuli, including microbes, extracellular crystals, pore-forming toxins, reactive oxygen species (ROS), and cathepsin B (9). Upon NLRP3 activator stimulation, NLRP3 binds to apoptosis-associated speck-like protein (ASC) and caspase-1 to form a multiprotein complex called the NLRP3 inflammasome (10). The NLRP3 inflammasome regulates innate immunity and inflammation in response to infectious microbes and damaged tissue (10).

Full activation of the NLRP3 inflammasome requires both priming and activation signals. Activation of TLR-4 by lipopolysaccharide (LPS) is one kind of priming signal that induces the expression of NLRP3 and interleukin (IL)-1β precursor (proIL-1β) (11). After priming, activation signals induce potassium efflux, mitochondrial damage, lysosomal rupture and chloride efflux, which all lead to NLRP3 inflammasome assembly and activation (12, 13). Activation of the NLRP3 inflammasome triggers caspase-1 activation and leads to the maturation of pro-inflammatory IL-1β and IL-18 (8, 10). It has been reported that human monocytes infected with NG produced IL-1β through NLRP3 inflammasome-dependent pathways, and this effect was suppressed by a cathepsin B inhibitor (14); however, the detailed mechanisms of how NG regulates the NLRP3 inflammasome are unclear. Understanding how NG activates the NLRP3 inflammasome would help to reveal the inflammatory responses of gonorrhea and offer therapeutic strategies to treat infections. In this study, we infected mouse and human macrophages and human monocytes with NG to establish NG-mediated inflammation models and explore the underlying mechanisms of how NG regulates NLRP3 inflammasome activation.

## Materials and Methods

### Reagents

MCC950 was purchased from TargetMol (Wellesley Hills, MA). Phorbol myristate acetate (PMA), ammonium chloride (NH_4_Cl), chloroquine diphosphate (CQ), N-acetyl-cysteine (NAC), pyrrolidine dithiocarbamate (PDTC), potassium chloride (KCl), glybenclamide, probenecid, carbenoxolone and cyclosporine A were purchased from Sigma-Aldrich (St. Louis, MO). Manganese (III) tetrakis (4-benzoic acid) porphyrin chloride (MnTBAP) and antibodies against ASC, IL-18, TLR4, P_2_X_7_, and actin were obtained from Santa Cruz Biotechnology (Santa Cruz, CA). PD98059, SB203580, SP600125, and antibodies against phospho-extracellular signal-regulated kinases 1/2 (ERK1/2), phospho-c-Jun N-terminal kinases 1/2 (JNK1/2), phospho-p38 and human caspase-1 were obtained from Cell Signaling Technology (Beverly, MA). Antibodies against NLRP3 and mouse caspase-1 were purchased from Adipogen International (San Diego, CA). Antibodies against IL-1β were purchased from R&D Systems (Minneapolis, MN). 10panx was purchased from Tocris Bioscience (Bristol, UK). The pHrodo Green *E. coli* BioParticles Conjugate was purchased from Thermo Fisher Scientific (Waltham, MA).

### Cell Culture

Mouse J774A.1 macrophages and human THP-1 monocytes were purchased from the American Type Culture Collection (Rockville, MD). To induce monocytes to differentiate into macrophages, THP-1 monocytes were incubated for 48 h with 100 nM PMA as described previously (15). Bone marrow-derived macrophages (BMDMs) were differentiated from bone marrow collected from C57BL/6 mouse femur and tibia. Human peripheral blood mononuclear cells (PBMCs) were isolated from whole blood by Ficoll-Hypaque density gradient centrifugation method as described previously (15). TLR2-, TLR4-, and P_2_X_7_-knockdown J774A.1 macrophages were generated by TLR2 shRNA lentiviral particles (Santa Cruz, catalog number: sc-40257-V), TLR4 shRNA lentiviral particles (Santa Cruz, catalog number: sc-40261-V), and P_2_X_7_ shRNA plasmids (Santa Cruz, catalog number: sc-42576-SH), respectively, and selected by puromycin. Control shRNA lentiviral particles (Santa Cruz, catalog number: sc-108080) and control shRNA plasmids (Santa Cruz, catalog number: sc-108060) were used as mock controls. NLRP3-knockout J774A.1 macrophages were generated by Cryopyrin CRISPR/Cas9 KO plasmids (Santa Cruz, catalog number: sc-432122). After transfection the CRISPR/Cas9 plasmids, the cells were growth individually in the 96-well-plate and 18 single cell clones were selected for NLRP3 protein expression analysis. One clone with significantly reduced NLRP3 protein expression was selected for further studies.

### Activation of NLRP3 Inflammasome by NG Infection

A standard *N. gonorrhoeae* strain (ATCC 49226) was purchased from the American Type Culture Collection (Rockville, MD). Cells were infected with NG at different multiplicities of infection (MOIs). After incubation of the infected cells at 37°C in 5% CO_2_ for 3 h, extracellular bacteria were killed by adding 1.6 μg/ml gentamicin to the culture medium. The cells were harvested at different time points as indicated. The expression levels of IL-1β, IL-18, and tumor necrosis factor-α (TNF-α) in the culture medium of NG-infected cells were measured using enzyme-linked immunosorbent assay (ELISA) kits (IL-1β and TNF-α kits from Affymetrix eBioscience, San Diego, CA; IL-18 kit from Elabscience, Houston, Texas) as described in detail previously (11). To detect the expression levels of proIL-1β/IL-1β, proIL-18/IL-18, p45/p10, NLRP3, and ASC in the culture medium, 300 μl culture medium was mixed with 300 μl methanol and 125 μl chloroform. After vortexing, 300 μl double-distilled water was added to the mixture, and it was incubated for 10 min on ice before centrifugation for 10 min at 13,000 rpm at 4°C. The supernatant was removed, and 500 μl methanol was added. After vortexing, the mixture was centrifuged for 10 min at 13,000 rpm at 4°C, and the supernatant was removed again. The pellet was dried at 55°C and dissolved in Western blotting loading buffer followed by incubation in boiling water for 30 min. The sample was further analyzed by Western blotting as described in detail previously (11). To detect the expression levels of proIL-1β, NLRP3, and actin and the phosphorylation levels of ERK1/2, JNK1/2, and p38 in the cells, the cell lysates from NG-infected cells were analyzed by Western blotting.

### Nuclear Factor-Kappa B (NF-κB) Reporter Assay

NF-κB reporter cells (J-Blue cells) were derived from J774A.1 macrophages with chromosomal integration of a secreted embryonic alkaline phosphatase (SEAP) reporter plasmid (pNiFty2-SEAP from InvivoGen, Carlsbad, CA). J-Blue cells were incubated for 30 min with or without NAC (10 mM) and then infected for 24 h with or without NG. The medium (20 μl) from the infected J-Blue cells was mixed with 200 μl QUANTI-Blue medium (Invitrogen) in 96-well-plates and incubated for 15 min at 37°C. SEAP activity was assessed by measuring the optical density at 655 nm using a microplate absorbance reader.

### Intracellular ROS Detection

Intracellular ROS production was detected by measuring the fluorescence intensity of 2′,7′-dichlorofluorescein, the oxidation product of 2′,7′-dichlorofluorescein diacetate (H_2_DCFDA) (Molecular Probes, Eugene, OR). Briefly, J774A.1 macrophages were incubated for 30 min with or without NAC (10 mM) followed by incubation for 30 min with 2 μM H_2_DCFDA and then infected with NG at 50 MOI for 4 h. The fluorescence intensity of 2′,7′-dichlorofluorescein was detected with an excitation wavelength of 485 nm and an emission wavelength of 530 nm using an iMark™ Microplate Absorbance Reader (Bio-Rad Laboratories Inc., Hercules, CA, USA).

### Mitochondrial Dysfunction Detection

To detect mitochondrial ROS production, J774A.1 macrophages and THP-1 monocytes were infected for 4 h with NG at 50 and 20 MOI, respectively. The cells were stained with 5 nM MitoSOX (Thermo Scientific, Rockford, IL) for 15 min. The fluorescence signal was analyzed by flow cytometry (Cytomics FC500 Flow Cytometry CXP, Beckman Coulter Life Sciences). To detect the mitochondrial inner transmembrane potential and mitochondrial mass, J774A.1 macrophages and THP-1 monocytes were infected for 4 h with NG at 50 and 20 MOI, respectively. The cells were stained with 25 nM MitoTracker Deep Red arnd MitoTracker Green (Thermo Scientific, Rockford, IL) for 15 min. The fluorescence signal was analyzed by flow cytometry.

### RT and Quantitative Real-Time PCR Analysis

RNA from wild-type (mock), TLR2-, TLR4-, or P_2_X_7_-knockdown macrophages were reverse transcribed prior to quantitative PCR analysis using the StepOne real-time PCR system (Applied Biosystems, Foster City, CA). TLR2 and TLR4 mRNA expression data was presented as the relative expression normalized to that of glyceraldehyde-3-phosphate dehydrogenase (GAPDH). The primers used were the following: TLR2, forward: CGCCCTTTAAGCTGTGTCTC; TLR2, reverse: CGATGGAATCGATGATGTTG; TLR4, forward: 5′-CCTGATGACATTCCTTCT-3′; TLR4, reverse: 5′-AGCCACCAGATTCTCTAA-3′; P_2_X_7_, forward: TGGAACCCAAGCCGACGTTGA; P_2_X_7_, reverse: CTCGGGCTGTCCCCGGACTT; GAPDH, forward: 5′-TGAAGGGTGGAGCCAAAAGG-3′; GAPDH, reverse: 5′-GATGGCATGGACTGTGGTCA-3′.

### Phagocytosis and Bactericidal Activity Assay

For 4 h infection, wild-type and NLRP3-knockout J774A.1 macrophages were infected with NG at 50 MOI. After incubating for 4 h, the extracellular bacteria were removed by washing the cells three times with PBS. The cells were then incubated in PBS containing 10 μg/ml gentamicin for 2 h on ice to further eliminate the adherent extracellular bacteria. For 8 h infection, wild-type and NLRP3-knockout J774A.1 macrophages were infected with NG at 50 MOI. After incubating for 4 h, the extracellular bacteria were removed by washing the cells three times with PBS, and followed by incubated in the culture medium containing 1.6 μg/ml gentamicin for an additional 4 h in the cell incubator. The cells were lysed by sterile H_2_O and the number of viable bacteria within the cells was determined by counting the colony-forming units. For phagocytosis assay, wild-type and NLRP3-knockout J774A.1 macrophages were incubated with pHrodo Green *E. coli* BioParticles Conjugate (2 μg) for 1 h. The intracellular fluorescence was measured by flow cytometry.

### Statistical Analysis

GraphPad Prism 7.0 software was used for data analysis. Data are shown as mean ± SD. Statistical significance was determined by *t*-tests (two-tailed) for two groups or ANOVA (with Dunnett's multiple comparisons test) for three or more groups. *P* < 0.05 were considered to be statistically significant.

## Results

### NG Infection Activates the NLRP3 Inflammasome in Mouse and Human Macrophages

**Figure 1:**
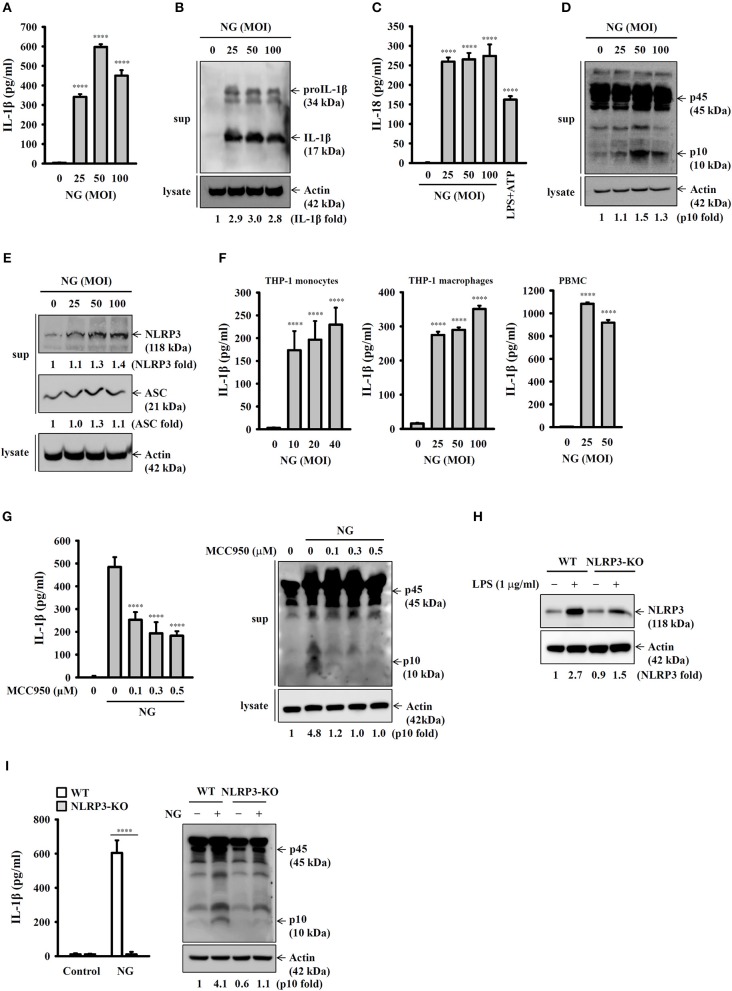
NG infection activates the NLRP3 inflammasome in mouse and human macrophages. **(A–E)** J774A.1 macrophages were infected for 24 h with NG. The levels of IL-1β and IL-18 in the supernatants were measured by ELISA **(A,C)**, and the levels of proIL-1β/IL-1β **(B)**, p45/p10 **(D)**, and NLRP3/ASC **(E)** in the supernatants were measured by Western blotting analysis. **(F)** THP-1 monocytes, THP-1 macrophages and PBMCs were infected for 24 h with NG. The levels of IL-1β in the supernatants were measured by ELISA. **(G)** J774A.1 macrophages were incubated for 0.5 h with MCC950 followed by infected for 24 h with NG. The levels of IL-1β and p45/p10 in the supernatants were measured by ELISA and Western blotting analysis, respectively. **(H)** Wild-type and NLRP3-knockout (NLRP3-KO) J774A.1 macrophages were incubated for 5 h with LPS (1 μg/ml). The protein expression levels of NLRP3 were measured by Western blotting. **(I)** Wild-type or NLRP3-KO J774A.1 macrophages were infected for 24 h with NG. The levels of IL-1β and p45/p10 in the supernatants were measured by ELISA and Western blotting analysis, respectively. The ELISA data are expressed as the mean ± SD of three separate experiments. The Western blotting results are representative of three different experiments, and the band intensity was analyzed by ImageJ and expressed as the fold change compared with the control group normalized to actin. A significant difference at the level of ^****^p < 0.0001 compared to untreated control cells **(A,C,F)**, NG-infected cells **(G)** or as indicated **(I)**. [One-way ANOVA with Dunnett's multiple comparisons test in **(A,C,F,G)**; two-tailed *t-*test in **(I)**].

To investigate whether NG infection activates the NLRP3 inflammasome, mouse J774A.1 macrophages were infected with NG at 25, 50, and 100 MOIs for 24 h. We found that the level of IL-1β in the culture medium was increased by NG infection by ELISA (Figure 1A) and Western blotting (Figure 1B). NG infection also increased the level of IL-18 (Figure 1C) and active caspase-1 (p10) (Figure 1D) in the culture medium analyzed by ELISA and Western blotting, respectively, confirming the activation of the NLRP3 inflammasome in NG-infected J774A.1 macrophages. It has been demonstrated that upon activation of the NLRP3 inflammasome, NLRP3 and ASC will be released from macrophages, acting as an extracellular danger signal and amplifying the inflammatory response (16). We found that NG infection induced NLRP3 and ASC release into the culture medium (Figure 1E). In addition, NG infection also increased the levels of IL-1β in the culture medium of human THP-1 monocytes, human THP-1 macrophages, and human PBMCs compared to the corresponding uninfected cells (Figure 1F). To investigate whether the IL-1β secretion and caspase-1 activation by NG is mediated by NLRP3 inflammasome, we performed the NG-mediated IL-1β secretion and caspase-1 activation in MCC950-treated J774A.1 macrophages. The results showed that NG-mediated IL-1β secretion and caspase-1 activation were impaired in MCC950-treated cells compared to the DMSO (vehicle)-treated cells (Figure 1G). In addition, to provide the direct evidence for NG-mediated IL-1β secretion through NLRP3 inflammasome, we generated the NLRP3-knockout J774A.1 by Cryopyrin CRISPR/Cas9 KO plasmids. We found that LPS-mediated NLRP3 expression was significantly reduced in NLRP3-knockout cells compared to the wild-type cells (Figure 1H). Although the NLRP3-knockout cells were generated by single cell clone, there is residual NLRP3 protein expression in the cells, indicating that only one allele was targeted. We demonstrated that NG-induced IL-1β secretion and caspase-1 activation were impaired in NLRP3-knockout cells compared to the wild-type cells (Figure 1I). These data indicated that the IL-1β secretion and the caspase-1 activation induced by NG is mediated by NLRP3 inflammasome.

### Activation of the NLRP3 Inflammasome by Live NG Through Lysosomal Acidification

**Figure 2:**
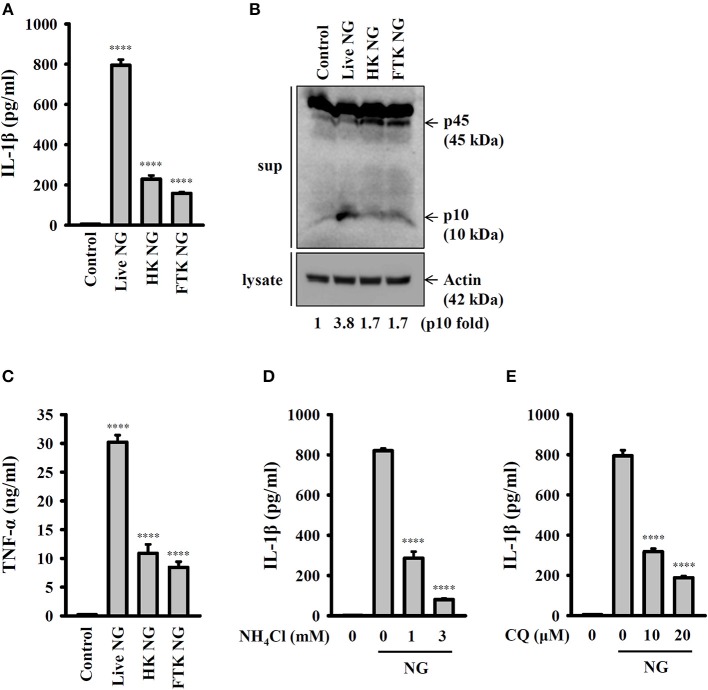
Activation of the NLRP3 inflammasome by live NG through lysosomal acidification. **(A–C)** J774A.1 macrophages were infected for 24 h with live, heat-killed (HK) or freeze/thaw-killed (FTK) NG at 50 MOI. The levels of IL-1β **(A)** and TNF-α **(C)** in the supernatants were measured by ELISA, and the levels of p45/p10 in the supernatants were measured by Western blotting analysis **(B)**. **(D,E)** J774A.1 macrophages were incubated for 0.5 h with NH_4_Cl **(D)** or CQ **(E)**, followed by infection for 24 h with NG at 50 MOI. The levels of IL-1β in the supernatants were measured by ELISA. The ELISA data are expressed as the mean ± SD of three separate experiments. The Western blotting results are representative of three different experiments, and the band intensity was analyzed by ImageJ and expressed as the fold change compared with the control group normalized to actin. A significant difference at the level of ^****^p < 0.0001 compared to untreated control cells **(A,C)** or NG-infected cells **(D,E)**. (One-way ANOVA with Dunnett's multiple comparisons test).

To further investigate whether activation of the NLRP3 inflammasome requires live NG, the induction of IL-1β and active caspase-1 in response to live, heat-killed or freeze/thaw-killed NG was investigated in J774A.1 macrophages. We found that live NG induced IL-1β secretion (Figure 2A) and caspase-1 activation (Figure 2B) significantly; however, these effects were significantly reduced in response to heat-killed and freeze/thaw-killed NG. In addition, killed NG also induced lower levels of TNF-α compared to that induced by live NG (Figure 2C). These results suggested that full activation of the NLRP3 inflammasome required live NG. In addition, it has been shown that the inhibition of lysosomal cysteine protease cathepsin B by Ca-074-me attenuates IL-1β secretion and cell death in NG-infected human THP-1 monocytes (14), suggesting an important role for the lysosome in NG-mediated NLRP3 inflammasome activation. As cathepsin B can be released from damaged lysosomes to drive NLRP3 inflammasome activation by binding to NLRP3 (17), NH_4_Cl and CQ, which both inhibit endosomal/lysosomal acidification, were used to block lysosomal acidification. We found that both NH_4_Cl (Figure 2D) and CQ (Figure 2E) significantly reduced IL-1β secretion in NG-infected J774A.1 macrophages, confirming a role for lysosomes in NLRP3 inflammasome activation in response to NG infection.

### Priming of the NLRP3 Inflammasome by Live NG Through TLR2

**Figure 3:**
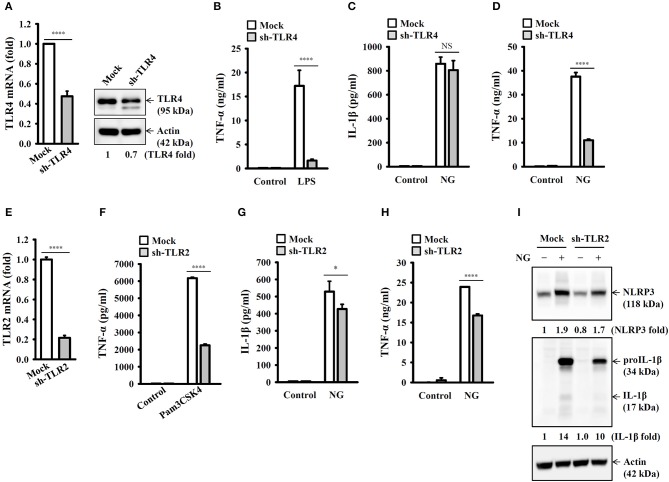
Priming of the NLRP3 inflammasome by live NG through TLR2. **(A)** The mRNA and protein expression levels of TLR4 in wild-type (mock) or TLR4-knockdown (sh-TLR4) J774A.1 macrophages were measured by RT real-time PCR and Western blotting, respectively. **(B)** Mock or sh-TLR4 cells were incubated for 6 h with 1 μg/ml LPS. The levels of TNF-α in the supernatants were measured by ELISA. **(C,D)** Mock or sh-TLR4 cells were infected for 24 h with NG at 50 MOI. The levels of IL-1β **(C)** and TNF-α **(D)** in the supernatants were measured by ELISA. **(E)** The mRNA expression levels of TLR2 in mock or TLR2-knockdown (sh-TLR2) J774A.1 macrophages were measured by RT real-time PCR. **(F)** Mock or sh-TLR2 cells were incubated for 6 h with 1 μg/ml Pam3CSK4. The levels of TNF-α in the supernatants were measured by ELISA. **(G–I)** Mock or sh-TLR2 cells were infected for 24 h **(G,H)** or 8 h **(I)** with NG at 50 MOI. The levels of IL-1β and TNF-α in the supernatants were measured by ELISA **(G,H)**, and the levels of NLRP3 and proIL-1β/IL-1β in the cell lysates were measured by Western blotting **(I)**. The RT real-time PCR and ELISA data are expressed as the mean ± SD of three separate experiments. The bands intensity of Western blotting was analyzed by ImageJ and expressed as the fold change compared with the mock control group normalized to actin. A significant difference at the level of ^*^p < 0.05 and ^****^p < 0.0001. NS, not significant (two-tailed *t*-test).

It has been reported that TLR4 played an important role in NG infection-mediated interferon-β expression in macrophages (18). To investigate whether TLR4 plays a role in NG-mediated NLRP3 inflammasome priming step, we generated the TLR4 knockdown J774A.1 macrophages by TLR4 shRNA lentiviral particles. We found that mRNA and protein expression levels of TLR4 were significantly reduced in TLR4-knockdown cells compared to the mock cells (Figure 3A). The LPS-mediated TNF-α secretion was impaired in TLR4-knockdown cells compared to the mock cells, confirming the functional knockdown of TLR4 in the cells (Figure 3B). Notably, TLR4-knockdown did not significantly affect NG-mediated IL-1β secretion (Figure 3C); however, the NLRP3 inflammasome-independent TNF-α secretion was significantly reduced (Figure 3D). These results indicated that priming of the NLRP3 inflammasome by NG was independent of TLR4. TLR2 can be activated by NG in T cells (19), suggesting the possible involvement of TLR2 in NG-mediated NLRP3 inflammasome priming step in macrophages. We generated the TLR2 knockdown J774A.1 macrophages by TLR2 shRNA lentiviral particles. We found that mRNA expression level of TLR2 was significantly reduced in TLR2-knockdown cells compared to the mock cells (Figure 3E). The Pam3CSK4-mediated TNF-α secretion was impaired in TLR2-knockdown cells compared to the mock cells, confirming the functional knockdown of TLR2 in the cells (Figure 3F). TLR2-knockdown reduced NG-mediated secretion of IL-1β (Figure 3G) and TNF-α (Figure 3H) compared to mock cells. In addition, NG-mediated NLRP3 and proIL-1β expression were also reduced in TLR2-knockdown cell compared to the mock cells (Figure 3I). These results indicated that priming of the NLRP3 inflammasome by NG was through TLR2.

### Activation of the NLRP3 Inflammasome by NG Through NF-κB- and Mitogen-Activated Protein Kinase (MAPK)-Dependent Pathways

**Figure 4:**
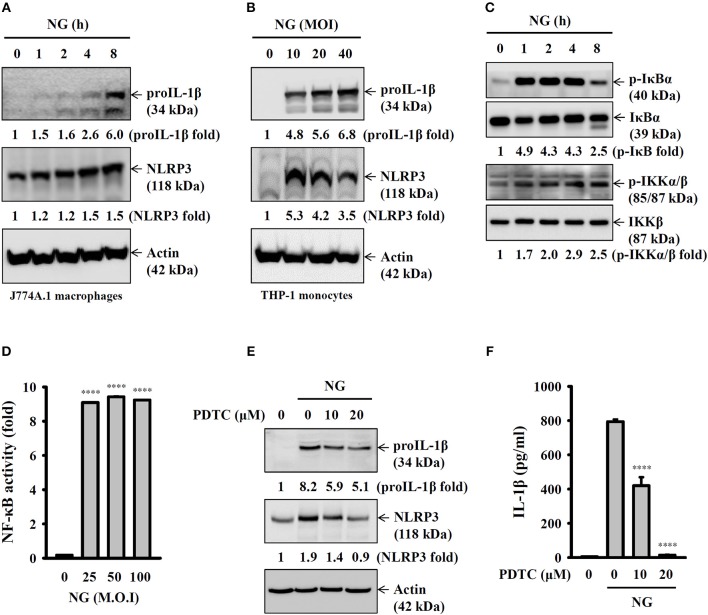
Activation of the NLRP3 inflammasome by NG through NF-κB-dependent pathways. **(A–C)** J774A.1 macrophages were infected for 0–8 h with NG at 50 MOI **(A,C)** or THP-1 monocytes were infected for 8 h with NG **(B)**. The levels of proIL-1β, NLRP3, p-IκBα/IκBα, and p-IKKα/β/IKKβ in the cell lysates were measured by Western blotting analysis. **(D)** J-Blue cells were infected for 24 h with NG. The activation of NF-κB was measured by QUANTI-Blue medium. **(E)** J774A.1 macrophages were incubated for 0.5 h with PDTC, followed by infection for 8 h with NG at 50 MOI. The levels of proIL-1β and NLRP3 in the cell lysates were measured by Western blotting analysis. **(F)** J774A.1 macrophages were incubated for 0.5 h with PDTC, followed by infection for 24 h with NG at 50 MOI. The levels of IL-1β in the supernatants were measured by ELISA. The ELISA data are expressed as the mean ± SD of three separate experiments. The Western blotting results are representative of three different experiments, and the band intensity was analyzed by ImageJ and expressed as the fold change compared with the control group normalized to actin or corresponding total protein. A significant difference at the level of ^****^p < 0.0001 compared to untreated control cells **(D)** or NG-infected cells **(F)**. (One-way ANOVA with Dunnett's multiple comparisons test).

**Figure 5:**
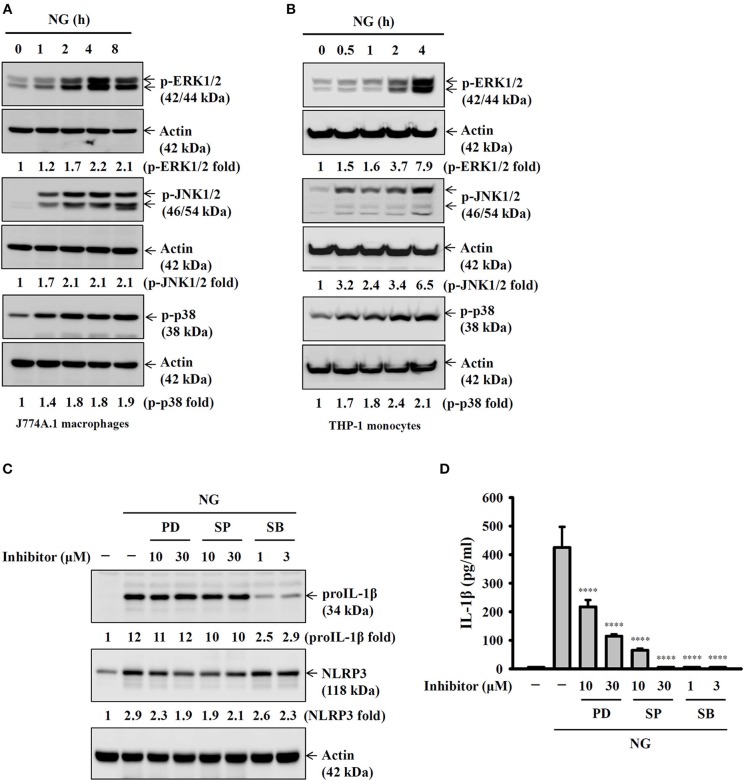
Activation of the NLRP3 inflammasome by NG through MAPK-dependent pathways. **(A,B)** J774A.1 macrophages were infected for 0–8 h with NG at 50 MOI **(A)** or THP-1 monocytes were infected for 0-4 h with NG at 20 MOI **(B)**. The amounts of phosphorylated ERK1/2, JNK1/2, and p38 in the cell lysates were measured by Western blotting analysis. **(C)** J774A.1 macrophages were incubated for 0.5 h with PD, SP or SB, followed by infection for 8 h with NG at 50 MOI. The levels of proIL-1β and NLRP3 in the cell lysates were measured by Western blotting analysis. **(D)** J774A.1 macrophages were incubated for 0.5 h with PD, SP or SB, followed by infection for 24 h with NG at 50 MOI. The levels of IL-1β in the supernatants were measured by ELISA. The ELISA data are expressed as the mean ± SD of three separate experiments. The Western blotting results are representative of three different experiments, and the band intensity was analyzed by ImageJ and expressed as the fold change compared with the mock control group normalized to actin. A significant difference at the level of ^****^p < 0.0001 compared to NG-infected cells (One-way ANOVA with Dunnett's multiple comparisons test).

Full activation of the NLRP3 inflammasome requires priming and activation signals, the former controlling the expression of NLRP3 and proIL-1β and the latter controlling the activation of caspase-1 (20). We found that NG infection increased the expression levels of NLRP3 and proIL-1β in J774A.1 macrophages (Figure 4A) and human THP-1 monocytes (Figure 4B). In a mechanistic study, we investigated the effect of NG infection on NF-κB activation by detecting the phosphorylation levels of IκBα and IKKα/β in J774A.1 macrophages. We found that the phosphorylation levels of IκBα and IKKα/β were significantly increased by NG infection (Figure 4C). The effect of NG infection on NF-κB activation was confirmed, as NG infection was shown to activate NF-κB transcriptional activity as assayed by an NF-κB reporter assay (Figure 4D). In addition, inhibition of NF-κB by PDTC reduced the expression levels of NLRP3 and proIL-1β (Figure 4E) and IL-1β secretion (Figure 4F) in NG-infected J774A.1 macrophages. NG infection also increased the phosphorylation levels of MAPKs (ERK1/2, JNK1/2, and p38) in J774A.1 macrophages (Figure 5A) and human THP-1 monocytes (Figure 5B). Inhibition of p38 by SB203580 significantly reduced proIL-1β expression in NG-infected J774A.1 macrophages; however, inhibition of ERK1/2 and JNK1/2 by PD98059 and SP600125, respectively, did not affect proIL-1β expression (Figure 5C). These results indicated that NG infection induced proIL-1β expression through p38-dependent pathways. Additionally, inhibition of ERK1/2 and JNK1/2 by PD98059 and SP600125, respectively, significantly reduced NLRP3 expression; however, inhibition of p38 by SB203580 did not affect NLRP3 expression (Figure 5C). These results indicated that NG infection induced NLRP3 expression through ERK1/2- and JNK1/2-dependent pathways. As MAPK inhibitors reduced proIL-1β or NLRP3 expression, NG-mediated IL-1β secretion was also suppressed by these inhibitors (Figure 5D). These results indicated that NG infection induced the priming signal of the NLRP3 inflammasome through NF-κB- and MAPK-dependent pathways.

### Activation of the NLRP3 Inflammasome by NG Through ROS-Dependent Pathways

**Figure 6:**
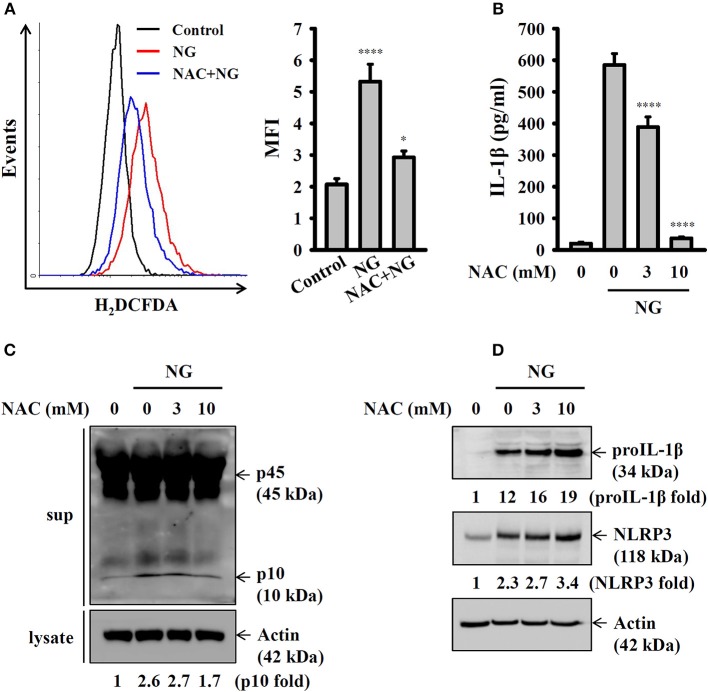
Activation of the NLRP3 inflammasome by NG through ROS-dependent pathways. **(A)** J774A.1 macrophages were incubated for 0.5 h with 10 mM of NAC, followed by infection for 4 h with NG at 50 MOI. The levels of ROS in the cells were measured by 2′,7′-dichlorofluorescein diacetate. **(B,C)** J774A.1 macrophages were incubated for 0.5 h with NAC, followed by infection for 24 h with NG at 50 MOI. The levels of IL-1β in the supernatants were measured by ELISA **(B)**, and the levels of p45/p10 in the supernatants were measured by Western blotting analysis **(C)**. **(D)** J774A.1 macrophages were incubated for 0.5 h with NAC, followed by infection for 8 h with NG at 50 MOI. The levels of proIL-1β and NLRP3 in the cell lysates were measured by Western blotting analysis. The ROS and ELISA data are expressed as the mean ± SD of three separate experiments. The Western blotting results are representative of three different experiments, and the band intensity was analyzed by ImageJ and expressed as the fold change compared with the mock control group normalized to actin. A significant difference at the level of ^*^p < 0.05 and ^****^p < 0.0001, respectively, compared to untreated control cells **(A)** or NG-infected cells **(B)** (One-way ANOVA with Dunnett's multiple comparisons test).

Previous studies demonstrated that generation of ROS is one of the crucial elements for NLRP3 inflammasome activation (21). In this study we found that NG infection increased ROS production in J774A.1 macrophages, and this effect was reduced by the antioxidant NAC (Figure 6A). Inhibition of ROS by NAC reduced IL-1β secretion (Figure 6B) and caspase-1 activation (Figure 6C) in NG-infected J774A.1 macrophages. However, NAC did not reduce the expression level of NLRP3 or proIL-1β in NG-infected J774A.1 macrophages (Figure 6D). These results indicated that ROS participated in the activation signal but not the priming signal of the NLRP3 inflammasome in NG-infected J774A.1 macrophages.

### Activation of the NLRP3 Inflammasome by NG Through P_2_X_7_ Receptor-Mediated K^+^ Efflux

**Figure 7:**
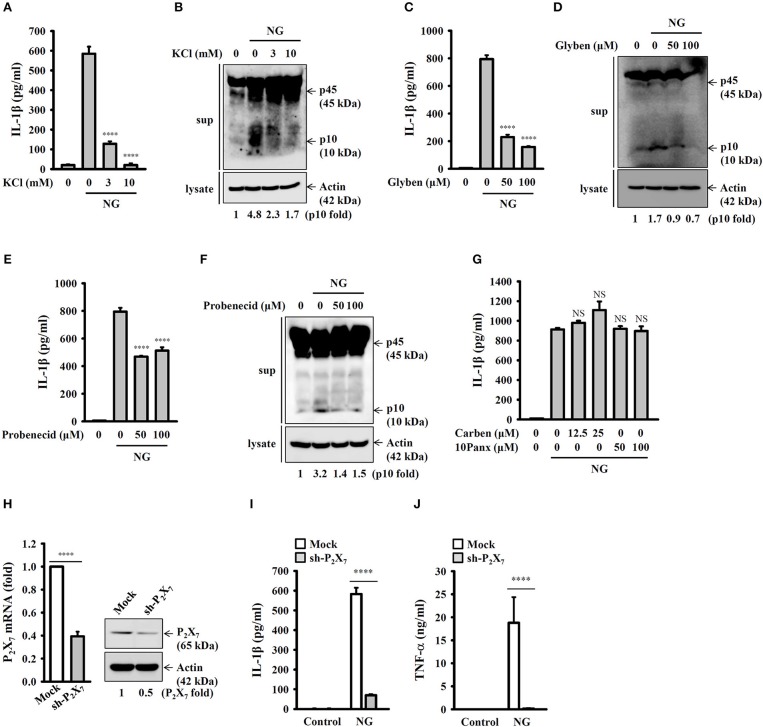
Activation of the NLRP3 inflammasome by NG through P_2_X_7_ receptor-mediated K^+^ efflux. J774A.1 macrophages were incubated for 0.5 h with KCl **(A,B)**, glybenclamide (Glyben) **(C,D)**, probenecid **(E,F)**, or carbenoxolone (Carben) and 10Panx **(G)**, followed by infection for 24 h with NG at 50 MOI. The levels of IL-1β in the supernatants were measured by ELISA, and the levels of p45/p10 in the supernatants were measured by Western blotting analysis. **(H)** The mRNA and protein expression levels of P_2_X_7_ in wild-type (mock) or P_2_X_7_-knockdown (sh-P_2_X_7_) J774A.1 macrophages were measured by RT real-time PCR and Western blotting, respectively. **(I,J)** Mock or sh-P_2_X_7_ cells were infected for 24 h with NG at 50 MOI. The levels of IL-1β **(I)** and TNF-α **(J)** in the supernatants were measured by ELISA. The ELISA data are expressed as the mean ± SD of three separate experiments. The Western blotting results are representative of three different experiments, and the band intensity was analyzed by ImageJ and expressed as the fold change compared with the mock control group normalized to actin. A significant difference at the level of ^****^p < 0.0001 compared to NG-infected cells **(A,C,E)** or as indicated **(H–J)**. NS, not significant [One-way ANOVA with Dunnett's multiple comparisons test in **(A,C,E)**; two-tailed *t*-test in **(H–J)**].

It has been demonstrated that K^+^ efflux is a common trigger of the NLRP3 inflammasome induced by various NLRP3 stimulators (22). To investigate whether K^+^ efflux is involved in NG-induced activation of the NLRP3 inflammasome, a high extracellular K^+^ concentration (3 and 10 mM KCl in culture medium) was used to test if inhibiting K^+^ efflux can influence NG-mediated activation of the NLRP3 inflammasome. We found that high extracellular KCl significantly reduced IL-1β secretion (Figure 7A) and caspase-1 activation (Figure 7B) in NG-infected J774A.1 macrophages. The role of K^+^ efflux on NG-mediated activation of the NLRP3 inflammasome was confirmed by glybenclamide, an adenosine triphosphate (ATP)-sensitive K^+^ channel blocker. We found that blocking K^+^ efflux with glybenclamide reduced IL-1β secretion (Figure 7C) and caspase-1 activation (Figure 7D) in NG-infected J774A.1 macrophages. To further identify the receptor involved in the K^+^ efflux, we tested the effect of a P_2_X_7_ receptor inhibitor (probenecid) and pannexin-1 inhibitors (carbenoxolone and 10Panx) on NG-mediated NLRP3 inflammasome activation. We found that probenecid reduced IL-1β secretion (Figure 7E) and caspase-1 activation (Figure 7F) in NG-infected J774A.1 macrophages; however, carbenoxolone and 10Panx did not affect IL-1β secretion significantly (Figure 7G). To provide the direct evidence for P_2_X_7_-mediated NLRP3 inflammasome activation in NG-infected macrophages, we generated the P_2_X_7_ knockdown J774A.1 macrophages by P_2_X_7_ shRNA plasmids. The P_2_X_7_ mRNA and protein expressions were reduced in P_2_X_7_-knockdown cells compared to the control (mock) cells (Figure 7H). We found that NG-induced IL-1β secretion was impaired in P_2_X_7_-knockdown cells compared to the mock cells (Figure 7I). These results indicated that NG infection activates the NLRP3 inflammasome through P_2_X_7_ receptor. Notably, NG-mediated TNF-α secretion was also impaired in P_2_X_7_-knockdown cells compared to the mock cells (Figure 7J).

### Activation of the NLRP3 Inflammasome by NG Through Mitochondrial Damage

**Figure 8:**
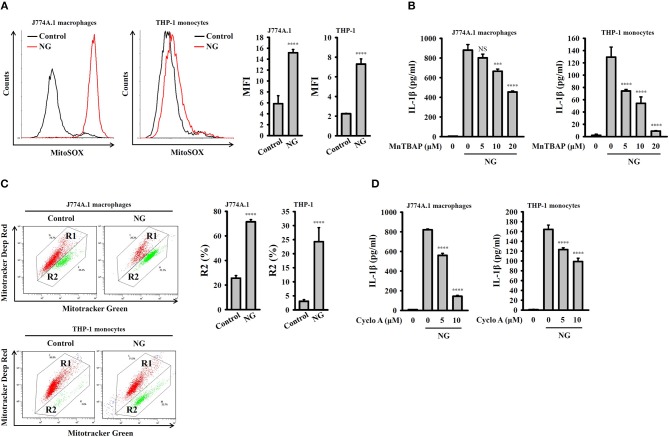
Activation of the NLRP3 inflammasome by NG through mitochondrial damage. **(A)** J774A.1 macrophages or THP-1 monocytes were infected for 4 h with NG at 50 or 20 MOI, respectively. The mitochondrial ROS levels in the cells were measured by staining with MitoSOX. **(B)** J774A.1 macrophages or THP-1 monocytes were incubated for 0.5 h with MnTBAP, followed by infection for 24 h with NG at 50 or 20 MOI, respectively. The levels of IL-1β in the supernatants were measured by ELISA. **(C)** J774A.1 macrophages or THP-1 monocytes were infected for 4 h with NG at 50 or 20 MOI, respectively. The mitochondrial inner transmembrane potential and mitochondrial mass were measured by staining with MitoTracker Deep Red and MitoTracker Green, respectively. **(D)** J774A.1 macrophages or THP-1 monocytes were incubated for 0.5 h with cyclosporine A (Cyclo A), followed by infection for 24 h with NG at 50 or 20 MOI, respectively. The levels of IL-1β in the supernatants were measured by ELISA. The flow cytometry and ELISA data are expressed as the mean ± SD of three separate experiments. A significant difference at the level of ^***^p < 0.001 and ^****^p < 0.0001, respectively, compared to untreated control cells **(A,C)** or NG-infected cells **(B,D)**. NS, not significant (One-way ANOVA with Dunnett's multiple comparisons test).

K^+^ efflux induced by NLRP3 stimulators activated mitochondrial ROS production and reduced the integrity of the mitochondria, which drove downstream signaling leading to NLRP3 inflammasome activation (23, 24). To investigate whether mitochondrial ROS are involved in NG-induced activation of the NLRP3 inflammasome, the mitochondrial ROS indicator MitoSOX was used to test whether NG infection can induce mitochondrial ROS production. We found that NG infection significantly induced mitochondrial ROS production in J774A.1 macrophages and human THP-1 monocytes (Figure 8A). Inhibition of mitochondrial ROS production by manganese (III) tetrakis (4-benzoic acid) porphyrin chloride (MnTBAP), which mimics mitochondrial superoxide dismutase (13), inhibited IL-1β secretion in NG-infected J774A.1 macrophages and human THP-1 monocytes (Figure 8B). In addition, using Mitotracker Deep Red and Mitotracker Green double staining, we found that the integrity of the mitochondria was reduced by NG infection in J774A.1 macrophages and human THP-1 monocytes (Figure 8C). Treatment with cyclosporine A, an inhibitor of the mitochondrial membrane permeability transition (21), reduced IL-1β secretion in NG-infected J774A.1 macrophages and in human THP-1 monocytes (Figure 8D). These results suggested the importance of mitochondrial ROS production and mitochondrial integrity loss in NG-mediated NLRP3 inflammasome activation.

### NLRP3 Knockout Increases the Bactericidal Activity of Macrophages Against NG

**Figure 9:**
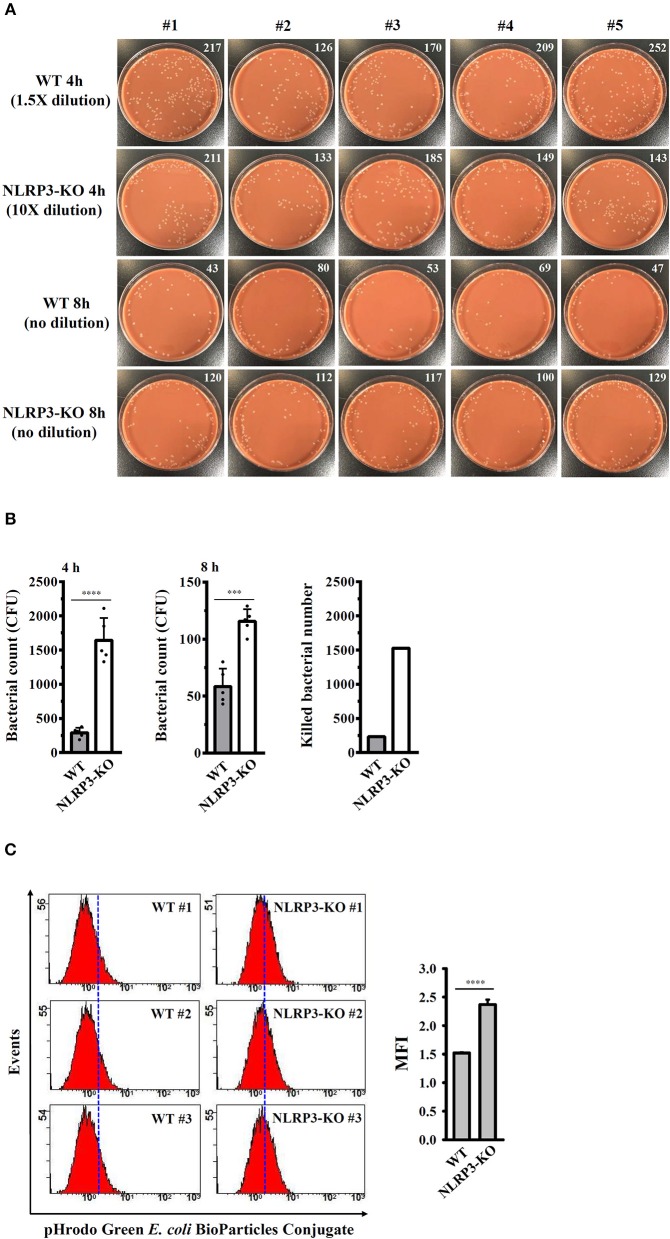
NLRP3 knockout increases the bactericidal activity of macrophages against NG. **(A,B)** Wild-type and NLRP3-knockout (NLRP3-KO) J774A.1 macrophages were infected with NG for 4 h and 8 h. The cells were lysed, and the number of engulfed live NG was determined by CFU assay and indicated in the upper right corner. The number of killed bacteria was calculated by subtracting the 8 h CFU assay result from the 4 h CFU assay result. **(C)** Wild-type and NLRP3-KO J774A.1 macrophages were incubated with pHrodo Green *E. coli* BioParticles Conjugate (2 μg) for 1 h. The intracellular fluorescence was measured by flow cytometry. The data are expressed as the mean ± SD of five **(B)** or three **(C)** separate experiments. A significant difference at the level of ^***^p < 0.001 and ^****^p < 0.0001, respectively (Two-tailed *t*-test).

The effects of the NLRP3 inflammasome on phagocytosis and the bactericidal activity of J774A.1 macrophages were further investigated. Wild-type and NLRP3-knockout J774A.1 macrophages were infected with NG, and the number of engulfed bacteria was determined 4 h after infection by colony-forming unit (CFU) assay. As shown in Figures 9A,B, the number of engulfed bacteria in NLRP3-knockout cells (1642 ± 146 CFU) was higher than that in wild-type cells (292 ± 32 CFU). These results indicated that NLRP3-knockout increased the phagocytosis of NG by macrophages. In addition, we measured the CFUs after 8 h of infection and found that the CFU in NLRP3-knockout and wild-type cells were 116 ± 5 and 58 ± 7, respectively (Figures 9A,B). This indicated that there were approximately 1,526 and 234 bacteria (calculated by subtracting the 8 h mean CFU from the 4 h mean CFU) killed in the NLRP3-knockout and wild-type cells, respectively, within 4 h (Figure 9B). To confirm the role of the NLRP3 in the phagocytosis activity of macrophages, the phagocytosis of pHrodo Green *E. coli* BioParticles Conjugate by macrophages were performed. We found that intracellular fluorescence in NLRP3-knockout cells was higher than that in wild-type cells (Figure 9C). These results indicated that NLRP3 knockout increases the phagocytosis and bactericidal activity of macrophages against NG.

## Discussion

Activation of the NLRP3 inflammasome is an important host defense response that limits microbial invasion (25, 26); however, deregulated activation of the NLRP3 inflammasome is associated with the pathogenesis of inflammatory diseases, including multiple sclerosis, Alzheimer's disease, Parkinson's disease, atherosclerosis, type 2 diabetes, and obesity (10). Thus, activation of the NLRP3 inflammasome should be tightly controlled to limit microbial invasion but prevent extensive inflammatory responses that are potentially hazardous to the host during infections (27, 28). Understanding how pathogens activate the NLRP3 inflammasome may provide insight into the mechanisms of host defense against microbes and is essential for developing potential treatment approaches against pathogenic infections. In this study, we dissected the signaling pathways that NG uses to manipulate macrophage inflammation by stimulating NLRP3 inflammasome-mediated IL-1β and IL-18 production. We demonstrated that NG infection induces caspase-1 activation by providing both priming and activation signals to the NLRP3 inflammasome, leading to IL-1β, and IL-18 production. Notably, NG infection at 100 MOI induced less IL-1β secretion and caspase-1 activation compared to 50 MOI. We suggested that NG infection at high MOI may cause severe cell death (29, 30), which leads to less IL-1β secretion and caspase-1 activation. However, the actual mechanism need further investigation.

Previous study indicated that NG (P9-17 strain) induced IL-1β mRNA expression in human blood monocyte-derived macrophage; however, P9-17 strain did not induce IL-1β secretion. Notably, exogenous ATP treatment induced IL-1β secretion from P9-17 strain-infected cells (31). In addition, ATP-mediated IL-1β secretion did not through increasing the caspase-1 activation in P9-17 strain-infected cells, but may act through the vesicle trafficking or pore formation levels (31). These results were conflicting with our and other findings, as NG strains: ATCC 49226 (this study), 1291 (32) and FA1090 (14) induced IL-1β secretion without additional ATP treatment. Another interesting finding is that lipooligosaccharide from wild-type NG (strain 1291 with hexa-acylated lipid A; activate TLR4), but not lipooligosaccharide from msbB-deficient strain 1291 NG (penta-acylated lipid A; did not activate TLR4) induced IL-1β secretion in human THP-1 monocytes and mouse bone marrow-derived macrophage in the presence of ATP (32), suggesting that lipooligosaccharide from wild-type NG provided the priming signal of NLRP3 inflammasome (e.g., increase NLRP3 and proIL-1β expression). In addition, although msbB-deficient NG at low MOI (1 MOI) induced less levels of IL-1β and TNF-α than wild-type NG, wild-type or msbB-deficient NG at higher MOI (10 MOI) induced similar levels of IL-1β and TNF-α (32). These results suggested that higher MOI infection may activate additional signaling pathways leading to the IL-1β and TNF-α secretion, that could compensate for the loss of TLR4-mediated signal pathways. Notably, although msbB-deficient NG did not activate TLR4 signaling, it activates caspase-1 similar to the wild-type NG, indicating that TLR4 is not necessary for NG-mediated caspase-1 activation (30, 32). These results support our current findings that TLR4 is not necessary for NG-mediated IL-1β secretion at high MOI infection (Figure 3C). However, one of the limitations of this study is the pathogen associated molecular patterns of NG in response to NLRP3 inflammasome activation has not been found.

It has been showed that Opa^+^ NG (strain MS11) induced gene expression levels of IL-1β and NLRP3 in neutrophil, and also increased the IL-1β secretion; however, the signaling pathways in the regulation of IL-1β and NLRP3 were not addressed yet (33). We are the first time to demonstrated that NG infection induced NLRP3 and proIL-1β expression through NF-κB activation, indicating that NF-κB is one of the important priming signal of the NLRP3 inflammasome in NG-infected macrophages. These findings are consistent with previous studies that NG infection activates NF-κB activation in epithelial cells (34–36). In addition, previous study demonstrated that NG (strain N400) activates ERK1/2, JNK1/2 and p38 in T84 human colonic epidermoid cells. The activated ERK1/2, JNK1/2, and p38 regulated the expression of stress-responsive genes and protected the cells from apoptosis (37, 38). In this study we demonstrated that NG induced NLRP3 expression through ERK1/2 and JNK1/2, while induced proIL-1β expression through p38 (Figure 5C).

Increasing data have shown that the NLRP3 inflammasome is activated in macrophages in response to various bacterial, viral, and fungal infections. Both gram-positive and gram-negative bacteria can induce NLRP3 inflammasome activation. The gram-positive bacteria *Vibrio cholera* (39), *Chlamydia pneumonia* (40), and *Shigella Flexneri* (41) activated the NLRP3 inflammasome in macrophages. Other gram-negative bacteria, such as *Staphylococcus aureus* (42), *Streptococcus pyogenes* (43), *Mycobacterium marinum* (44), *Mycobacterium tuberculosis* (45), *Listeria monocytogenes* (46), enterohemorrhagic *Escherichia coli*, and *Citrobacter rodentium* (47), also induced activation of the NLRP3 inflammasome in macrophages. In addition, NG infection promoted NLRP3 inflammasome activation in monocytes through activation of the lysosomal protease cathepsin B (14); however, the detailed signaling pathways induced by NG infection for the regulation of NLRP3 inflammasome activation are unclear. In this study, we further demonstrated that endosomal/lysosomal acidification plays a crucial role in NG-mediated NLRP3 inflammasome activation, as NH_4_Cl and CQ significantly suppressed IL-1β secretion (Figures 2F,G). Lysosomal rupture and cathepsin B release are also important for NLRP3 inflammasome activation in response to *Histoplasma capsulatum* (48) and *Mycobacterium kansasii* infection (25).

The activation signals for the NLRP3 inflammasome may come from a variety of PAMPs sor damage-associated molecular patterns (DAMPs) (12). The NLRP3 inflammasome is activated by numerous PAMPs during bacterial, fungal and viral infections (28), including nigericin, a pore forming toxin derived from *Streptomyces hygroscopicus* (42). The NLRP3 inflammasome-activating DAMPs include metabolic danger signals (i.e., cholesterol crystals, uric acid crystals, saturated fatty acids, and islet amyloid polypeptide) and amyloid-β in Alzheimer's disease (10). Several cellular signals have been proposed as activation signals for the NLRP3 inflammasome, including K^+^ efflux, ROS production, mitochondrial damage, and lysosomal rupture (12). We demonstrated that NG-mediated activation of the NLRP3 inflammasome was suppressed by high extracellular levels of KCl and the ATP-sensitive K^+^ channel blocker glybenclamide, indicating the important role of K^+^ efflux (Figures 7A–D). It has been demonstrated that phagocytosis of particulate matter, i.e., aluminum hydroxide, silica, calcium pyrophosphate crystals and L-leucyl-L-leucine methyl ester, triggers K^+^ efflux and induces NLRP3 inflammasome activation (22). In this study, we found that IL-1β secretion and caspase-1 activation were significantly reduced in heat-killed NG-treated macrophages compared to live NG-infected macrophages (Figures 2A,B). These results suggested that phagocytosis of live NG is required for K^+^ efflux and NLRP3 inflammasome activation. Previous study also showed that internalization of live NG (strain FA1090B) specifically induced pyroptosis and IL-1β secretion in human monocyte-derived macrophages, and these effects were due in part to intracellular lipooligosaccharide (30).

It has been suggested that rapid K^+^ efflux through ATP-activated P_2_X_7_ receptor (49) and membrane channel pannexin-1 (50) induces NLRP3 inflammasome assembly and activation in neutrophils and gingival epithelial cells, respectively. Another study demonstrated that probenecid directly blocked the P_2_X_7_ receptor and reduced ATP-mediated IL-1β secretion in LPS-primed human monocytes and J774A.1 macrophages; however, pannexin-1 inhibitor carbenoxolone and the pannexin-1 mimetic inhibitory peptide, 10Panx did not affect IL-1β secretion (51). Our study showed the similar results that NG induced NLRP3 inflammasome activation through P_2_X_7_ receptor, but not pannexin-1, as the IL-1β secretion was significantly reduced by probenecid and P_2_X_7_ receptor shRNA, but not reduced by carbenoxolone and 10Panx (Figures 7E–I). Notably, NG-mediated TNF-α secretion was also significantly reduced in P_2_X_7_-knockdown J774A.1 macrophages (Figure 7J). Recent study demonstrated that P_2_X_7_ plays important roles in TNF-α converting enzyme activation, the key enzyme controlling TNF-α secretion (52). K^+^ efflux triggers downstream signaling and leads to mitochondrial damage, thereby activating the NLRP3 inflammasome (23, 24). Mitochondrial damage is characterized by mitochondrial ROS production and the loss of the mitochondrial inner transmembrane potential (23, 24). Mitochondrial ROS promote the translocation of intracellular chloride channels to the plasma membrane for the induction of chloride efflux to promote NLRP3 inflammasome assembly, caspase-1 activation and IL-1β secretion (13). It has been demonstrated that porin protein (PorB) secreted from NG (MS11-A strain) via outer membrane vesicles targeted to mitochondria of macrophages, which leads to the loss of mitochondrial membrane potential and caspase-dependent cell death (29). These results agree with our current findings that NG infection caused mitochondrial damage as evidenced by mitochondrial ROS production and the loss of mitochondrial integrity (Figures 8A,C). Scavenging mitochondrial ROS by MnTBAP or preserving mitochondrial integrity by cyclosporine A suppressed IL-1β secretion in NG-infected macrophages and monocytes (Figures 8B,D). We suggested that NG infection may cause chloride efflux and induce the physical interactions between NLRP3 and NEK7 (53), NLRP3 and ASC (54), and NLRP3 and PKR (55), leading to the activation of the NLRP3 inflammasome; however, the detailed mechanisms require further investigation.

**Figure 10:**
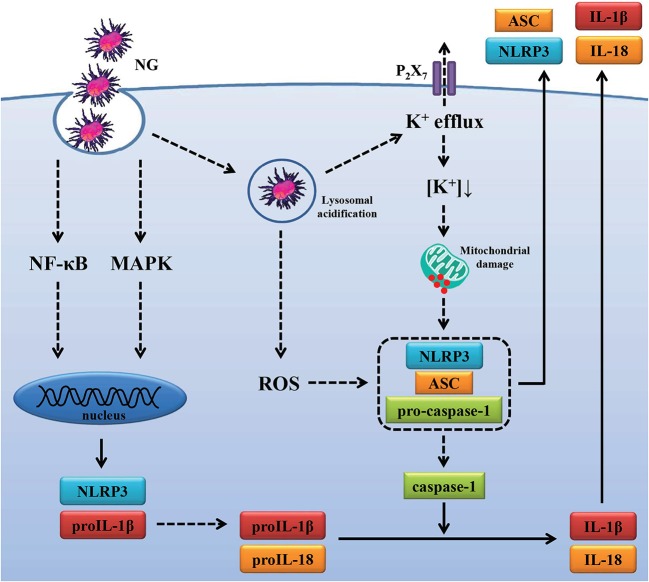
Overview of the putative mechanisms by which NG activated the NLRP3 inflammasome. NG activated the NLRP3 inflammasome in macrophages through (1) increasing NLRP3 and proIL-1β expression by activating NF-κB and MAPK; (2) increasing caspase-1 activation and IL-1β/IL-18 secretion by ROS production, lysosomal acidification, P_2_X_7_ receptor-mediated K^+^ efflux and mitochondrial damage.

Activation of the NLRP3 inflammasome is required for host defense against bacterial infections (25, 56–59); however, the beneficial antimicrobial role of the NLRP3 inflammasome must be tightly regulated to prevent pathogenic effects induced by excess inflammation. Mice lacking NLRP3 were more susceptible than wild-type mice to infection with *M. kansasii* (25), group B *Streptococcus* (56), *C. rodentium* (57), *Burkholderia pseudomallei* (58), and *Streptococcus pneumonia* (59). In contrast, Knockout of NLRP3 protects mice from lethal *S. aureus* pneumonia by increased bactericidal function of macrophages (60). In addition, although the NLRP3 inflammasome contributes to the immunopathology of tuberculosis induced by *M. marinum* and *M. tuberculosis* infection, it was not associated with bacterial growth in infected mice (44, 61). Previous study found that the NLRP3 inflammasome is required for IL-1β and IL-18 production in NG-infected macrophages (14). In this study we demonstrate that NLRP3 knockout increased the phagocytosis activity and the bactericidal activity of macrophages against NG (Figure 9). However, the role of the NLRP3 inflammasome in the host defense against NG *in vivo* needs further study. In conclusion, our results indicated that NG infection induced NLRP3 inflammasome activation through ROS/NF-κB-, MAPK-, K^+^ efflux-, lysosomal rupture- and mitochondrial damage-dependent pathways (Figure 10). In theory blocking only one pathway would not be sufficient to suppress NG-mediated responses; however, blocking one of the pathways significantly reduced the NLRP3 inflammasome activation in NG-infected macrophages. These results suggested that these pathways may interact with each other in NG-infected macrophages, but the detailed interaction between these pathways need further investigation. These molecules, proteins and pathways participated in NG-mediated NLRP3 inflammasome activation can be the drug targets for the development of anti-inflammatory agents for the treatment of NG infections in the future.

## Data Availability

The raw data supporting the conclusions of this manuscript will be made available by the authors, without undue reservation, to any qualified research.

## Ethics Statement

The mice study was performed with the approval of the Institutional Animal Care and Use Committee of the National Ilan University (approval number: No. 106-13) according to the NIH Guide for the Care and Use of Laboratory Animals. PBMCs were isolated from whole blood of healthy volunteers recruited at the Tri-Service General Hospital in Taipei, Taiwan, after their approval according to the Institutional Review Board of the Tri-Service General Hospital, National Defense Medical Center and the patients' informed consent (approval number: TSGH-IRB-2-106-05-190 and TSGH-IRB-2-106-05-009). Bacterial infection was performed with the approval of Taiwan CDC (approval number: 098013).

## Author Contributions

L-HL and K-FH designed the experiments, analyzed the data, and wrote the manuscript. L-HL, J-SL, H-WC, W-YL, T-CJ, F-HC, OC, M-LL, and J-CC performed the experiments. C-HH, AC, S-MK, and H-WG contributed to critical revision of the manuscript.

### Conflict of Interest Statement

The authors declare that the research was conducted in the absence of any commercial or financial relationships that could be construed as a potential conflict of interest.
